# Supplementary material for: Identification of a Novel Germline PPP4R3A Missense Mutation Asp409Asn on Familial Non-Medullary Thyroid Carcinoma
Source: Biomedicines. 2024 Jan 22;12(1):244. doi: 10.3390/biomedicines12010244 (PMC10813271; doi:10.3390/biomedicines12010244)
Supplement: Supplementary file 1 [file biomedicines-12-00244-s001.zip › biomedicines-2663698-supplementary.pdf]

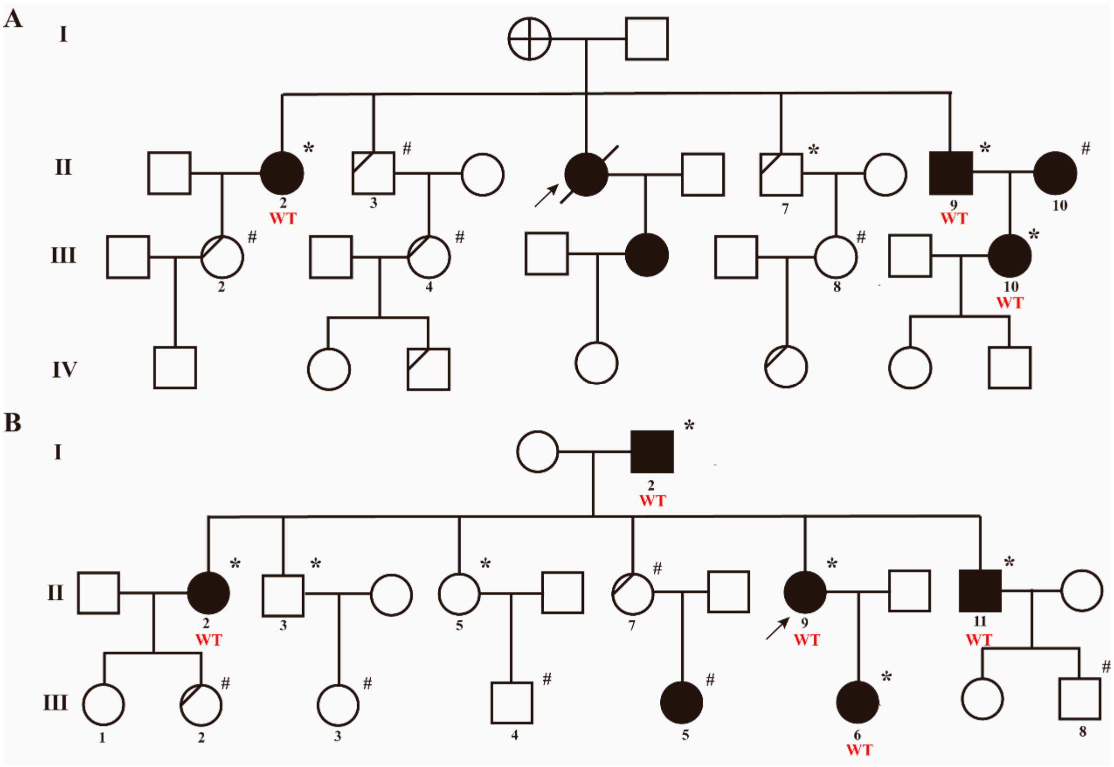

Supplementary Figure S1. Pedigree of two additional FNMTC families. Squares represent males, and circles represent females. Filled circles and squares represent diagnosed PTC patients. Short diagonal lines represent patients with thyroid nodules, and long diagonal lines represent deceased members. Squares with crossed lines indicate individuals with hyperthyroidism. The black arrow points to the FNMTC family proband. Subjects participating in this study are labeled with numbers. * denotes subjects analyzed by whole-exome sequencing; # denotes subjects analyzed by Sanger sequencing. "WT" stands for Wide Type, *PPP4R3A* wild type gene.

Table S1. Summary of shRNA sequences and primers used in this study.

PPP4R3A	Sh1	ACCGGTGCGTTTTGATGATATGTCACTCGAGTGAC ATATCATCAAAACGCTTTTTGAATTC	
	Sh2	ACCGGTGTTATGTTCTCTGAAGAATCTCGAGATTCT TCAGAGAACATAACTTTTTGAATTC	
	Sh3	ACCGGTCATACCTACCACATAAAGACTCGAGTCTT TATGTGGTAGGTATGTTTTTGAATTC	
PPP4R3A	Fwd	CAGGTGCGAAGTGCTGCTA	For genomic DNA
	Rev	GGCTACACTTTTCCCAAACAGC	
PPP4R3A	Fwd	GTTCTACCAACTCCTTCGGTCT	For mRNA
	Rev	TTCCTGCAACATGCCAACAAT	
GAPDH	Fwd	ACAACTTTGGTATCGTGGAAGG	
	Rev	GCCATCACGCCACAGTTTC	
